# The frequency of osteogenic activities and the pattern of intermittence between periods of physical activity and sedentary behaviour affects bone mineral content: the cross-sectional NHANES study

**DOI:** 10.1186/1471-2458-14-4

**Published:** 2014-01-06

**Authors:** Sebastien FM Chastin, Oleksii Mandrichenko, Dawn A Skelton

**Affiliations:** 1 School of Health and Life Science, Institute of Applied Health Research, Glasgow Caledonian University, Cowcaddens Road G4 0BA Glasgow, Scotland UK

**Keywords:** Sedentary behaviour, Osteoporosis, Bone mass, Physical activity, Accelerometry

## Abstract

**Background:**

Sedentary behaviours, defined as non exercising seated activities, have been shown to have deleterious effects on health. It has been hypothesised that too much sitting time can have a detrimental effect on bone health in youth. The aim of this study is to test this hypothesis by exploring the association between objectively measured volume and patterns of time spent in sedentary behaviours, time spent in specific screen-based sedentary pursuits and bone mineral content (BMC) accrual in youth.

**Methods:**

NHANES 2005–2006 cycle data includes BMC of the femoral and spinal region via dual-energy X-ray absorptiometry (DEXA), assessment of physical activity and sedentary behaviour patterns through accelerometry, self reported time spent in screen based pursuits (watching TV and using a computer), and frequency of vigorous playtime and strengthening activities. Multiple regression analysis, stratified by gender was performed on N = 671 males and N = 677 females aged from 8 to 22 years.

**Results:**

Time spent in screen-based sedentary behaviours is negatively associated with femoral BMC (males and females) and spinal BMC (females only) after correction for time spent in moderate and vigorous activity. Regression coefficients indicate that an additional hour per day of screen-based sitting corresponds to a difference of −0.77 g femoral BMC in females [95% CI: -1.31 to −0.22] and of −0.45 g femoral BMC in males [95% CI: -0.83 to −0.06]. This association is attenuated when self-reported engagement in regular (average 5 times per week) strengthening exercise (for males) and vigorous playing (for both males and females) is taken into account. Total sitting time and non screen-based sitting do not appear to have a negative association with BMC, whereas screen based sedentary time does. Patterns of intermittence between periods of sitting and moderate to vigorous activity appears to be positively associated with bone health when activity is clustered in time and inter-spaced with long continuous bouts of sitting.

**Conclusions:**

Some specific sedentary pursuits (screen-based) are negatively associated with bone health in youth. This association is specific to gender and anatomical area. This relationship between screen-based time and bone health is independent of the total amount of physical activity measured objectively, but not independent of self-reported frequency of strengthening and vigorous play activities. The data clearly suggests that the frequency, rather than the volume, of osteogenic activities is important in counteracting the effect of sedentary behaviour on bone health. The pattern of intermittence between sedentary periods and activity also plays a role in bone accrual, with clustered short bouts of activity interspaced with long periods of sedentary behaviours appearing to be more beneficial than activities more evenly spread in time.

## Background

Osteoporosis is a major public health issue gaining importance with the ageing of the population. Osteoporotic fractures are estimated to cost approximately $17.9 billion per year in the US and £1.7 billion in the UK [1].

Peak bone mass achieved as a youth is the strongest predictor of later life osteoporosis risk [2,3]. Peak bone mass is limited by genetics [4], but environmental and lifestyle factors influence successful achievement of this genetic potential [4]. The osteogenic effects of weight bearing physical activity (PA) and exercise have long been recognised [5]. There is now strong evidence that increased bone mass in childhood is associated with frequency, intensity and the type of PA [6-8]. It is thought that PA and exercise directly promote bone growth, not just directly during high impact activities but also indirectly by increasing muscle mass and strength, which creates more tension on skeletal structures [9].

Lack of engagement in PA has therefore been recognised as a risk factor for osteoporosis [10]. This lack of PA has often been referred to as ‘sedentary behaviour’ (SB) or a ‘sedentary lifestyle’ [11] and comes under the umbrella term ‘inactivity’. Recently, evidence has emerged from many lines of enquiry, including epidemiology and laboratory studies, showing that time spent sitting has specific deleterious effects on health independent of the PA levels [12]. SB is now regarded as a separate construct from inactivity and defined as time spent sitting or reclining [13].

Through an analogy with bed rest and weightlessness studies on bone health [14-17], it has been suggested that repeated exposure to sitting could have a direct effect on bone mass, through increased bone resorption and decreased stimulation of bone formation [18,19].

Long periods of reduced weight-bearing activity directly affects people who are forced to remain in bed [20] or in space [16] and might seem irrelevant for the majority of the population. However, modern societal and technological changes have dramatically increased the time spent in low impact and reduced weight-bearing postures in everyday life [21]. Epidemiological surveys report that young people between the age of 6 and 20 spend on average 40 to 60% of the day sitting [22], often in prolonged and uninterrupted bouts.

There is a dearth of information about the possible association between SBs and bone health. Only three studies have investigated this. In adults, it has been found that the time spent in sedentary work is associated with osteoporotic fracture risk [23]. Vicente-Rodríguez et al. [24] showed an increased risk of low whole body bone mineral content (BMC) in male adolescents who watched television (TV) for more than 3 hours/day. Similarly, the time spent sitting while studying has been found to be associated with reduced whole-body BMC in adolescent girls [25], however, this association was completely attenuated when engagement of PA was taken into account. This suggests that SB does not have specific deleterious effect on BMC, but instead, either contributes to the effect of inactivity or its effect can be compensated with an engagement in osteogenic PA. However, this same study showed that the time boys spent sitting browsing the internet was found to be associated with lower BMC, independent of levels of PA [25].

Therefore, more information about the potential effects of sitting in daily life on bone health is required to identify whether sitting is a specific and independent risk or simply that seated pursuits displace time spent being active. As sitting is more prevalent in modern lifestyles it is also important to understand whether the deleterious effects of sitting can be moderated by PA.

This study aimed to investigate whether the time spent, during childhood, in different SBs is associated with BMC at fracture prone anatomical sites (femur and lumbar spine). Dual-energy X-ray absorptiometry (DEXA), objectively measured SB and PA, self-reported sitting and TV viewing time data from the 2005–6 cycle of NHANES were examined to investigate whether associations between different SBs and BMC were independent of PA.

## Methods

### Study

The National Health and Nutrition Examination Survey (NHANES) is a cross-sectional study conducted annually by the National Center for Health Statistics, Centers for Disease Control (CDC). NHANES uses a complex, multistage probability design to obtain a representative sample of the USA civilian non-institutionalized population over a two year cycle. Details about the surveys and methods of NHANES are available from the CDC website (http://www.cdc.gov/nchs/nhanes.htm). The study of bone health and osteoporosis is one of the major aims of NHANES and measurement of bone mineral content via DEXA has been part of the survey since NHANES III. The data from NHANES III actually serves as a reference value for the diagnosis of osteoporosis [26]. Concurrent self-reported and objective measurements of PA and SB with accelerometry and DEXA are available for the 2003–4 and 2005–6 cycles. However, the NHANES DEXA data for the 2003–4 cycle contains a systematic and non-random pattern of missing data and the CDC released a set of imputed values for this cycle. For this analysis we elected to include only data free of imputation and concentrated on the 2005–6 cycle. The study complies with the Declaration of Helsinki, the National Center for Health Statistics Ethics Review Board approved the protocols, and written informed consent was obtained.

### Study sample

In the 2005–6 NHANES cycle, 2779 youths below the age of peak bone mass density (8–22 years old [26]) had valid DEXA measurement of the femur region, 2747 had valid DEXA measurement of the spine. Of these, 1679 youths had valid accelerometry data. In total, accelerometry, DEXA and covariate data for 1348 individuals (Males N = 671) were available to analyse femur BMC and 1340 individuals (Males N = 677) for spine BMC.

### Bone mineral content measurement

BMC of the proximal femur and the lumbar (L1-L4) spine were measured using DEXA. For the lumbar spine, only the total spine (L1-L4) BMC rather than individual vertebrae levels were examined, as recommended by the International Society for Clinical Densitometry [27]. The DEXA scans were performed with Hologic QDR-4500A fan-beam densitometers (Hologic, Inc., Bedford, Massachusetts), by trained and certified radiology technologists. The scans were analysed using the Hologic software, APEX v3.0, which has been shown to have good precision [28]. High level of quality was maintained throughout the data collection with a rigorous quality control protocol, including regular anthropomorphic phantom scans checks. Further details of the DEXA data acquisition protocol are described in the Body Composition Procedures Manual on the Centers for Disease Control NHANES website (http://www.cdc.gov/nchs/nhanes/nhanes2005-2006/current_nhanes_05_06.htm).

### Self reported measures of physical activity and sedentary behaviours

The NHANES 2005–6 dataset contains self-reported information about specific sedentary behaviours (TV watching and computer use) as well as information about frequency of muscle strengthening, vigorous exercising and/or playing. Daily average time spent watching TV and using computers was collected from a 30 day recall questionnaire on a 6 point ordinal scale (<1, 1, 2, 3, 4, > = 5 hrs/day). Similarly, participants were asked to report the number of muscle strengthening activities over a 30 day period and the number of times per week they exercised or played vigorously. For this study, an estimated average total daily time spent in front of a screen was calculated by summing the TV watching and computer use time.

### Objective physical activity and sedentary behaviour monitoring and data processing

All ambulatory participants to the 2005–6 cycle of NHANES were eligible and asked to wear an Actigraph accelerometer (Actigraph 7164; Actigraph, LLC, Fort Walton Beach, FLA). The Actigraph accelerometer is a small (5.1 × 4.1 × 1.5 cm), lightweight (0.4 kg) device worn on the hip that records acceleration information integrated as an activity count per epoch (here 1 minute), which provides an objective estimate of the intensity of bodily movement (particularly ambulatory locomotion). Thresholds obtained from calibration studies allow the relation of accelerometer counts per minute (cpm) to levels of physical activity intensity [22,29]. The accelerometer was worn for 7 days during waking hours (except for water-based activities). The devices were returned by mail to NHANES and data downloaded and checked to ascertain if the device was still calibrated. Further details on the objective physical activity protocol can be found on the NHANES website (http://www.cdc.gov/nchs/nhanes/nhanes2005-2006/PAXRAW_D.htm).

Accelerometry data were first screened to exclude data from monitors not in calibration and data identified by the Centers for Disease Control as not meeting the NHANES quality control. An automated program [22] was adapted and used to implement these quality control procedures and isolate time during which the device was not worn. The standard definition of non-wear time, from the Centers for Disease Control (intervals of at least 60 consecutive minutes of 0 cpm, with allowance for up to 2 min of observations of some limited movement (<50 cpm) within these periods) was adopted. Recorded days with at least 10 hours of continuous wear time, that did not contain spurious, excessively high, counts (>20 000 cpm) were considered as valid. Individuals with at least 5 valid days, including 1 weekend day, were included in the analyses, as suggested by current best practice recommendation in physical activity monitoring [30]. 10% of cases were excluded because the accelerometry data did not meet calibration and quality control criteria. A further 39% of cases were excluded because of non-wear time criteria, leaving 1679 valid accelerometry cases.

Each 1 minute epoch of accelerometry data was classified according to age specific calibration equations [22,29,31,32] as sedentary (SB) or moderate and vigorous intensity physical activity (MVPA). For each valid day contiguous epoch in the same class were aggregated into bouts of length equal to the number of contiguous epochs. From the accelerometry data, outcomes reflecting the volume of time spent in MVPA and SB, and the pattern of accumulation of sedentary time were collated.

Total daily time spent in SB and MVPA was obtained by summing the duration of all the bouts in each level for each day. The values were normalised to total wear time and averaged over the number of valid days to derive an estimate of the mean daily time spent in SB and MVPA. Volume is presented as a percentage of the day.

The proportion of SB spent in non-screen based activity was estimated by subtracting the estimated daily average total screen time from the objectively measured SB.

The pattern of time spent in SB was characterised using the distribution (histrogram) of sedentary bout duration, as previously described by Chastin and Granat [33]. The distribution is a power law characterised by a parameter α. A low α can be interpreted as a tendency to accumulate SB in longer continuous bouts and to engage in MVPA in a more sporadic way with bouts of MVPA tending to cluster in time [34]. A high α indicates a more broken up pattern of SB with periods of MVPA occurring more regularly through the day.

All processing was done using MATLAB R11b (Mathworks Ltd).

### Statistical data analysis

All analysis were carried out with SPSS version 18 (IBM, Chicago, IL). The analysis was stratified by gender, as bone mass and osteogenic response to PA is different in males and females. The associations between BMC and objectively measured SB, as well as time spent in specific sedentary behaviours (TV watching and computer use), was examined by multiple regression. We first modelled the relationship of pattern and time spent in SB (Model 1) with BMC of the femur and lumbar spine region. Model 1 was then adjusted for time spent in MVPA (Model 2), self reported muscle strengthening activity (Model 3) and self-reported vigorous exercise/play (Model 4) to determine if this association between SB and BMC is attenuated by PA.

All models were adjusted for known risk factors associated with a decrease in BMC; age, smoking, body mass index and ethnicity. We also looked at additional potential confounders; serum vitamin D in the blood, calcium intake (history of milk intake), parental history of osteoporosis diagnosis, alcohol intake over the last 12 months and specifically for females, the use of birth control and age of first menstruation. Models for males and females were adjusted differently to account only for factors that were significant predictors.

During modelling all continuous variables were checked for normality before being entered in the models, root square transformation was used to normalise frequency of strengthening activities and daily computer use, logarithmic transformation was used for frequency of vigorous play and exercise time, daily screen time and first menstrual cycle age in females. For each regression model, the linearity of the association between predictors and outcomes, as well as all other required data conditions, were examined. Multicollinearity between independent variables was checked by performing variance inflation tests (VIF) (VIF score greater than 10 indicate the presence of collinearity [35]).

## Results

Table 1 shows descriptive variables of the sample by gender. Significant differences were found between gender groups with males spending less total time sedentary but spending more time using computers than females. Males also engaged in more MVPA, and more frequent vigorous playing and strengthening exercises than females. There was no statistically significant difference between the femur and spine groups in terms of gender distribution (Chi^2^ test, p = 0.787), age (t-test, p = 0.905), time spent in SB (t-test, p = 0.830), time spent in MVPA (t-test, p = 0.803), screen time (Mann–Whitney test p = 0.904), frequency of vigorous play (Mann–Whitney test p = 0.856) and frequency of strength training activities (Mann–Whitney test p = 0.998).

**Table 1:** Descriptive characteristics of sample (average and [range])

**Variables**	***Male***	**Female**
Age *(year)*	14.0 [8 – 22]	13.6 [8 – 22]
Spinal BMC^a^*(g)*	46.4 [16.2 – 100.1]	43.5 [13.2 – 85.6]
Femoral BMC^a^*(g)*	31.7 [9.3 – 67.2]	24.9 [55.7 – 7.54]
BMI^b^*(kg/m*^*2*^*)*	22.0 [12.8 – 54.4]	22.5 [12.4 – 50.4]
Sedentary time *(%)*	52.6 [21.2 – 90.6]*	55.1 [14.8 – 79.1]*
MVPA^c^*(%)*	23.2 [0.0 – 51.0]*	20.4 [0.0 – 74.0]*
TV watching *(hours/day)*	3.3 [1 – 6]	3.2 [1 – 6]
Computer use *(hours/day)*	2.3 [0 – 6]*	2.0 [0 – 6]*
Frequency of vigorous playing/exercising *(number per week)*	6.1 [0 – 21]*	5.4 [0 – 21]*
Frequency of strengthening activities *(number per month)*	11.5 [0 – 180]*	7.0 [0 – 60]*

^a^Bone mineral content.

^b^Body mass index.

^c^Moderate and vigorous physical activity.

*Significant difference between gender (p <0.05) (ANOVA test performed for normally distributed variables and Mann - Whitney U test performed for non-normally distributed variables).

**Figure 1:**
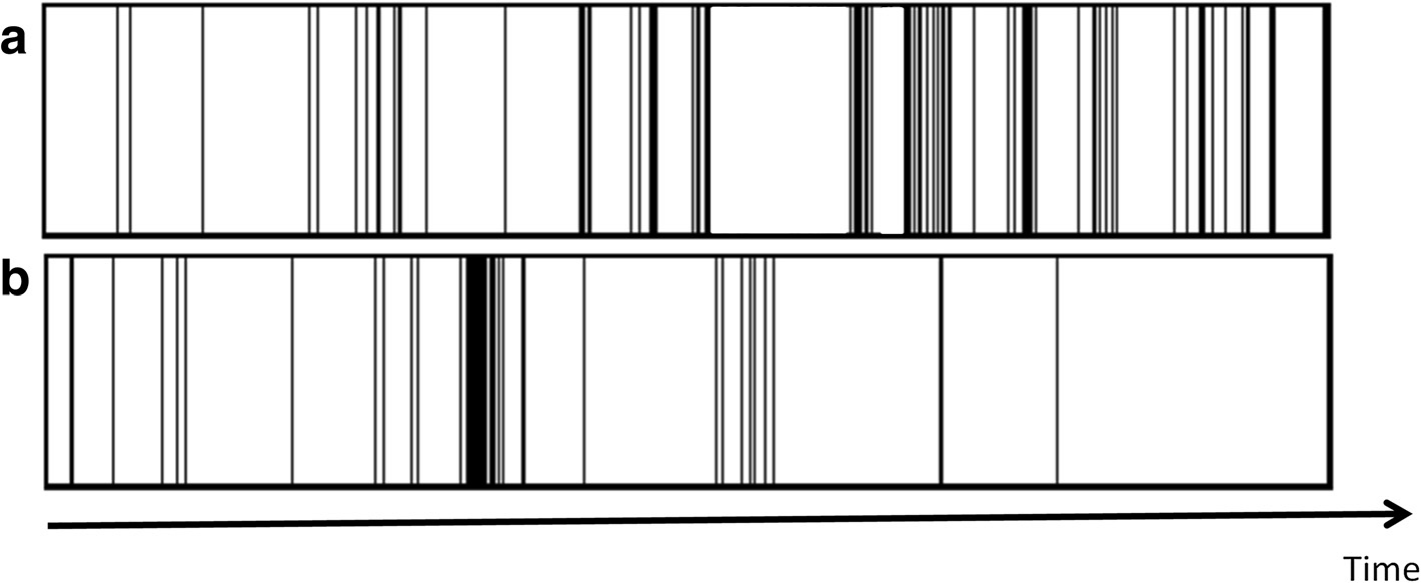
**Schematic representation of the temporal pattern of intermittence between periods of activity (black) and sedentary behaviours (white).** In pattern **a)** activity is distributed more evenly in time (high α) than in pattern **b)** where they are more clustered and inter-spaced with longer periods of sedentary behaviour (low α).

In males, time spent watching TV, and total screen-time, were negatively associated with femoral BMC (Table 2). This association was not attenuated by the introduction of objectively measured time spent in MVPA (Model 2), but disappeared when frequency of vigorous playing and strengthening exercise were introduced (Model 3 and 4). There was no association between total SB time and non screen based SB with femoral BMC, except in Model 2, for which total SB time and non screen based SB appear to be positively associated with femoral BMC. This is accompanied with a negative association between α, the marker of pattern of SB, and femoral BMC, indicating a negative effect of less clustered MVPA. Figure 1 illustrates two different patterns with equal amounts of MVPA. Pattern a) has few long periods of SB and periods of MVPA evenly distributed in time. It corresponds to a higher α. In pattern b) clustered periods of activity alternate with extended periods of rest. This pattern corresponds to a low α, which appears more beneficial to bone health. There was no association between objectively and subjectively measured SB and spinal BMC in males (Table 3), except for Model 2, which shows the same pattern of association as for femoral BMC.

**Table 2:** Multivariate association between sedentary behaviours and femoral BMC in men

**Model**	**Model R**^**2**^_**adj**_	**B (95% CI)**	**β**^**a**^
	**R**^**2 **^**change**		
**Model 1**	0.673		
Model adjusted for			
Age (β = 0.704)^***^, BMI (β = 0.231) ^***^, Ethnicity (β = 0.101)^***^ and Vitamin D (β = 0.076)^***^			
Objective sedentary time	0.000	−1.09 (−4.66 to 6.76)	0.009
Time spent watching TV	0.002^*^	−0.44 (−0.84 to −0.05)^*^	**−0.053**
Time spent on computer	0.001	−0.41 (−0.96 to 0.14)	−0.035
Total screen time	0.002^*^	−0.21 (−0.41 to 0.00)^*^	**−0.046**
Total non-screen SB	0.002	0.17 (−0.01 to 0.35)	0.044
α	0.000	−1.36 (−4.24 to 1.51)	−0.023
**Model 2**	0.687		
Model 1 adjusted for objectively measured MVPA (β = 0.144) ^***^			
Objective sedentary time	0.009^***^	15.93 (8.64 to 23.11)^***^	**0.143**
Time spent watching TV	0.002^*^	- 0.45 (−0.83 to −0.06)^*^	**−0.053**
Time spent on computer	0.000	−0.35 (−0.89 to 0.19)	−0.030
Total screen time	0.001	−0.82 (−1.80 to 0.16)	−0.039
Total non-screen SB	0.006^**^	0.327 (0.141 to 0.513)^**^	**0.095**
α	0.006^***^	−5.67 (−8.86 to −2.49)^***^	**−0.096**
**Model 3**	0.415		
Model 1 adjusted for weekly frequency of strengthening exercise (β = 0.102) ^**^			
Objective sedentary time	0.000	−1.26 (−8.37 to 5.85)	−0.013
Time spent watching TV	0.002	− 0.48 (−0.97 to 0.02)	−0.071
Time spent on computer	0.000	−0.41 (−1.21 to 0.34)	−0.038
Total screen time	0.003	−0.90 (−2.19 to 0.38)	−0.052
Total non-screen SB	0.001	0.13 (−0.11 to 0.36)	0.038
α	0.000	−0.27 (−0.40 to 0.34)	−0.005
**Model 4**^**e**^	0.510		
Model 1 adjusted for weekly frequency of vigorous playing or exercise (β = 0.126)^*^			
Objective sedentary time	0.002	−3.13 (−9.03 to 2.78)	−0.061
Time spent watching TV	0.001	0.114 (−0.28 to 0.47)	0.036
Time spent on computer	0.004	−0.24 (−0.60 to 0.12)	−0.076
Total screen time	0.000	0.12 (−0.71 to 0.96)	0.017
Total non-screen time SB	0.000	−0.03(−0.18 to 0.13)	−0.021
α	0.000	−1.88 (−7.15 to 3.40)	−0.08

^a^Standardised regression coefficients.

^*^Statistically significant predictor of BMC (p <0.05).

^**^Statistically significant predictor of BMC (p <0.01).

^***^Statistically significant predictor of BMC (p <0.0001).

Bold text highlights statistically significant associations.

**Table 3:** Multivariate association between sedentary behaviours and femoral BMC in women

**Model**	**Model R**^**2**^_**adj**_	**B (95% CI)**	**β**^**a**^
	**R**^**2 **^**change**		
**Model 1**	0.655		
Model adjusted for age (β = 0.377)^***^, age of first menstrual cycle (β = −0.236)^***^, BMI (β = 0.377)^***^ and Ethnicity (β = 0.086)^***^			
Objective sedentary time	0.000	0.21 (−3.67 to 4.19)	0.003
Time spent watching TV	0.003^*^	−0.28 (−0.50 to −0.06)^*^	**−0.057**
Time spent on computer	0.000	−0.18 (−0.54 to 0.19)	−0.022
Total screen time	0.004^**^	−0.80 (−1.35 to −0.25)^**^	**−0.066**
Total non-screen SB	0.004^**^	0.15 (0.04 to 0.26)^**^	**0.063**
α	0.001	−1.41 (−3.28 to 0.47)	−0.038
**Model 2**	0.662		
Model 1 adjusted for objectively measured MVPA (β = 0.130)^***^			
Objective sedentary time	0.005^***^	8.07 (3.18 to 12.97)^***^	**0.113**
Time spent watching TV	0.003^*^	- 0.26 (−0.48 to −0.05)^*^	**−0.055**
Time spent on computer	0.000	−0.16 (−0.52 to 0.20)	−0.020
Total screen time	0.004^**^	−0.77 (−1.31 to −0.22)^**^	**−0.063**
Total non-screen SB	0.006^**^	0.327 (0.141 to 0.513)^**^	**0.095**
α	0.007^***^	−4.05 (−6.18 to −1.92)^***^	**−0.109**
**Model 3**	0.415		
Model 1 adjusted for weekly frequency of strengthening exercise (β = 0.173)^***^			
Objective sedentary time	0.002	−3.21 (−8.25 to 1.81)	−0.049
Time spent watching TV	0.009^*^	− 0.33 (−0.59 to −0.07)^*^	−0.095
Time spent on computer	0.000	−0.10 (−0.56 to 0.35)	−0.017
Total screen time	0.009^*^	−0.86 (−1.54 to −0.17)^*^	−0.095
Total non-screen SB	0.004	0.11 (−0.02 to 0.25)	0.064
α	0.000	0.842 (−1.72 to 3.41)	0.025
**Model 4**	0.577		
Model 1 adjusted for weekly frequency of vigorous playing or exercise (β = 0.140)^**^			
Objective sedentary time	0.003	−3.15 (−7.85 to 1.55)	−0.061
Time spent watching TV	0.004	0.23 (−0.11 to 0.55)	0.065
Time spent on computer	0.000	−0.03 (−0.47 to 0.41)	−0.006
Total screen time	0.001	0.26 (−0.45 to 0.97)	0.034
Total non-screen time SB	0.000	−0.08 (−0.23 to 0.07)	−0.048
α	0.000	−0.513(−1.59 to 2.61)	0.022

^a^Standardised regression coefficients.

^*^Statistically significant predictor of BMC (p <0.05).

^**^Statistically significant predictor of BMC (p <0.01).

^***^Statistically significant predictor of BMC (p <0.0001).

Bold text highlights statistically significant associations.

In females, the results show significant negative associations between TV time and screen based SB with spinal and femoral BMC (Table 4). These associations are not changed or attenuated when time spent in MVPA or the frequency of strengthening activity was taken into account. However the frequency of vigorous playing/exercising appears to make these associations vanish (Model 4). As for males, objectively measured SB and estimated non screen based sedentary time were positively associated with BMC when total MVPA is taken into account. A pattern with less clustered MVPA separated by short periods of SB is negatively associated with BMC as illustrated by the results for α in Model 2.

**Table 4:** Multivariate association between sedentary behaviours and spinal BMC in men

**Model**	**Model R**^**2**^_**adj**_	**B (95% CI)**	**β**^**a**^
	**R**^**2 **^**change**		
**Model 1**	0.701		
Model adjusted for Age (β = 0.771)^***^, BMI (β = 0.153)^***^, Ethnicity (β = 0.094)^***^ and Vitamin D (β = 0.093)^***^			
Objective sedentary time	0.001	5.33 (−3.11 to 13.78)	0.031
Time spent watching TV	0.001	−0.47 (−1.06 to 0.12)	−0.036
Time spent on computer	0.001	−0.51 (−1.32 to 0.31)	−0.028
Total screen time	0.001	−1.17 (−2.60 to 0.25)	−0.037
Total non-screen SB	0.000	0.07 (−0.07 to 0.20)	0.021
α	0.002	−3.16 (−7.47 to 1.15)	−0.034
**Model 2**	0.708		
Model 1 adjusted for objectively measured MVPA (β = 0.097)^**^			
Objective sedentary time	0.009^***^	24.10 (13.22 to 34.99)^***^	**0.139**
Time spent watching TV	0.001	− 0.48 (−1.06 to 0.10) ^*^	−0.036
Time spent on computer	0.001	−0.45(−1.26 to 0.37)	−0.024
Total screen time	0.001	−1.08(−2.50 to 0.34)	−0.034
Total non-screen SB	0.001	0.04(−0.04 to 0.232)^**^	0.031
α	0.005^**^	−8.23 (−13.06 to −3.39) ^***^	**−0.090**
**Model 3**	0.482		
Model 1 adjusted for weekly frequency of strengthening exercise (β = 0.099)^**^			
Objective sedentary time	0.001	5.51 (−5.25 to 16.27)	0.036
Time spent watching TV	0.004	- 0.69 (−1.44 to 0.07)	−0.063
Time spent on computer	0.002	−0.84 (−2.06 to 0.39)	−0.047
Total screen time	0.004	−1.86 (−3.83 to 0.11)	−0.066
Total non-screen SB	0.004^*^	0.36 (0.00 to 0.72)^*^	**0.067**
α	0.002	−3.32(−8.99 to 2.36)	−0.040
**Model 4**	0.378		
Model 1 adjusted for weekly frequency of vigorous playing or exercise (β = 0.048)			
Objective sedentary time	0.000	−0.799 (−8.79 to 7.19)	−0.013
Time spent watching TV	0.005	0.276 (−0.21 to 0.76)	0.073
Time spent on computer	0.000	−0.06 (−0.55 to 0.43)	−0.016
Total screen time	0.001	0.27 (−0.46 to 0.99)	0.028
Total non-screen time SB	0.000	−0.01 (−0.05 to 0.07)	0.018
α	0.001	−0.78 (−4.39 to 2.84)	−0.028

^a^Standardised regression coefficients.

^*^Statistically significant predictor of BMC (p <0.05).

^**^Statistically significant predictor of BMC (p <0.01).

^***^Statistically significant predictor of BMC (p <0.001).

Bold text highlights statistically significant associations.

The results for spinal BMC (Table 5) are similar with TV watching and screen based SB appearing to have a negative association not modified by MVPA or frequency of strengthening exercises but attenuated by vigorous playing. As for femoral BMC in females and males, pattern (α) and volume of objectively measured SB are also associated with BMC only when MVPA is taken into account.

**Table 5:** Multivariate association between sedentary behaviours and spinal BMC in women

**Model**	**Model R**^**2**^_**adj**_	**B (95% CI)**	**β**^**a**^
	**R**^**2 **^**change**		
**Model 1**	0.643		
Model adjusted for age (β = 0.692)^***^, BMI (β = 0.197)^***^ and Ethnicity (β = 0.081)^**^			
Objective sedentary time	0.007^***^	14.13 (6.31 to 21.96)^***^	0.096^***^
Time spent watching TV	0.003^*^	−0.49 (−0.95 to 0.02)^*^	−0.049^*^
Time spent on computer	0.001	−0.53 (−1.29 to 0.29)	−0.032
Total screen time	0.005^**^	−0.40(−0.66 to -0.15)^**^	−0.073^**^
Total non-screen SB	0.010^***^	0.49(0.27 to 0.71)^***^	0.102^***^
α	0.006^**^	−6.74 (−10.62 to -2.86)^**^	−0.087^**^
**Model 2**	0.702		
Model 1 adjusted for objectively measured MVPA (β = 0.057)			
Objective sedentary time	0.008^***^	23.63 (12.91 to 34.35)^***^	0.137^***^
Time spent watching TV	0.001^*^	- 0.58 (−1.13 to -0.03)^*^	−0.044 ^*^
Time spent on computer	0.001	−0.61(−1.40 to 0.176)	−0.033
Total screen time	0.001	−0.08(−0.22 to 0.06)	−0.023
Total non-screen SB	0.013^***^	0.58(0.35 to 0.805)^***^	0.121^***^
α	0.013^***^	−11.24(−15.69 to -6.79)^***^	−0.146^***^
**Model 3**	0.276		
Model 1 adjusted for weekly frequency of strengthening exercise (β = 0.168)^***^			
Objective sedentary time	0.000	0.86 (−9.59 to 11.31)	0.007
Time spent watching TV	0.005	−0.46(−1.00 to 0.09)	−0.069
Time spent on computer	0.001	−0.30 (−1.26 to 0.65)	−0.026
Total screen time	0.007^*^	−0.34 (−0.65 to -0.03)^*^	−0.089^*^
Total non-screen SB	0.006	0.27 (−0.01 to 0.54)	0.080
α	0.000	0.03(−5.39 to 5.45)	0.000
**Model 4**	0.470		
Model 1 adjusted for weekly frequency of vigorous playing or exercise (β = 0.101)^*^			
Objective sedentary time	0.000	0.804 (−8.68 to 10.26)	0.009
Time spent watching TV	0.001	0.181 (−0.48 to 0.838)	0.030
Time spent on computer	0.001	−0.20 (−1.08 to 0.67)	−0.023
Total screen time	0.001	−0.08 (−0.39 to 0.24)	−0.066
Total non-screen time SB	0.001	0.08 (−0.21 to 0.37)	0.028
α	0.000	−0.72 (−4.95 to 3.51)	−0.028

^a^Standardised regression coefficients.

^*^Statistically significant predictor of BMC (p <0.05).

^**^Statistically significant predictor of BMC (p <0.01).

^***^Statistically significant predictor of BMC (p <0.001).

Bold text highlights statistically significant associations.

## Discussion

The main findings of this study are:

– some specific SB (TV watching and screen-based time) are negatively associated with BMC

these associations appear to be specific to anatomic sites and differ by gender

– the total amount of MVPA performed does not affect these associations

– engagement in frequent strengthening (in males) and vigorous exercising or playing activities (in males and females) appear to counteract the deleterious effects of SB

– there appears to be an intricate interplay between the amount of SB and the clustering patterns of physical activity.

These results suggest that sitting for extended periods of time during activities such as watching TV or using computers has a deleterious effect on bone health in children and young adults, and that excessive amounts of time spent in these activities might prevent young people from achieving their genetic peak bone mineral content potential. The results also suggest that engaging in vigorous playing and strengthening exercises frequently might counteract the deleterious effect of sitting on bone health. Interestingly, not all periods of sitting or the total volume of sitting appear detrimental. In fact, it appears that non screen based SB might have some beneficial impact on bone health when the pattern of intermittence between SB and MVPA is considered. This counter-intuitive result is consistent with several studies which have shown that osteogenic activities yield more bone mass increase when performed in short and clustered bursts rather than over regular sustained periods [36-39]. In addition, laboratory studies in rats have shown that the interpolation of rest periods is crucial to bone formation [40,41]. Perhaps some extended periods of SB in between bouts of osteogenic activities might be needed to allow the skeletal system physiology to adapt to osteogenic stimulation.

By analogy with bed rest and weightlessness studies, it has been hypothesised that too much sitting could have a negative effect on bone health [18]. This negative impact could stem from two distinct processes. First indirectly, as time spent in SB might displace engagement in physical activity, resulting in less osteogenic stimulation of the skeletal system. Secondly, repeated daily exposure to sitting for long periods might results in systemic physiological changes, affecting bone metabolism, similar to those observed in bed rest studies [15].

The results of this study appear to both support and challenge this hypothesis. As in the HELENA study [25], this study also found that only some specific SBs show negative associations with femoral BMC in youth, rather than the total SB time. This suggests that not all sitting time is detrimental and that the effect is localised to the lower limb. Therefore our results hint that the effect of sitting might not be systemic and that sitting does not have a direct physiological effect on bone health. Indeed, a systemic change in physiology induced by sitting should be proportional to the total time spent sitting. In this respect, the results seem to agree with multiple studies, which have shown that reducing sitting time by standing immobilised individuals does not affect femoral or spinal BMC [42-45]. In addition, if too much sitting changes bone metabolism systemically, one would expect this to affect both the femoral and spinal skeletal area, which does not seem to be the case.

However, in sitting only the lower limb muscles are unloaded but muscle tension on the spinal skeletal structure is increased [46]. According to the mechanostat theory [47], this might explain the anatomical differential effect of SB. Moreover, we found that the negative associations between SB and femoral BMC are independent of the total amount of MVPA performed. This suggests that sitting for extended periods of time has a deleterious effect on bone metabolism, independent of the volume of mechanical loading, hinting that this effect might be direct and systemic. It is, however, possible that the accelerometry actually underestimates the amount of osteogenic physical activity. Indeed most bouts of moderate and vigorous activity are very short and generally accelerometry results are reported on a 1 minute epoch basis. It is therefore possible that some epochs containing short bouts of activity are misclassified by the calibration equations resulting in an underestimate of the total time spent in moderate and vigorous activity.

This study has found that the frequency of strengthening exercise and vigorous playing completely attenuates the association between SB and BMC. Firstly, this suggests that if sitting has a negative effect on bone health it can be counteracted by promoting regular strengthening exercise and/or vigorous playing, without need for reducing total sitting time. Secondly, it reinforces the idea that sitting might not have a specific effect on bone metabolism, but that seated activities, such TV watching and computer use, displaces osteogenic activities. In this respect, sitting time should be a concern for bone health if leisure time seated activities draw children away from pursuits that involve osteogenic leisure activities.

Although our results are broadly similar to the only other available study on SB and bone health in children [25], there are some quantitative differences. Unlike the HELENA study, our study found the association of screen-based SB to be independent of time spent in MVPA but found the associations between SB and BMC to be less strong. This study uses the same objective measure of MVPA and procedures, so the differences could be attributed to the subjective measure of SB and sample size (larger in this study).

Collectively these two studies strongly suggest that classifying all seated activity under the umbrella term of sedentary behaviours [13] might not be helpful in understanding the effect of sitting time on health. In order to shed light on the impact of sitting on health, and particularly bone health, a more precise taxonomy of seated activity is required [48].

The strengths of this study are the use of objective measures of SB and PA and the quality of the dataset, in particular the quality control and calibration of all BMC and physical activity measures. However, screen time, frequency of vigorous play and exercise and strengthening activities were self-reported. Self-reported measures are less precise and tend to lead to underestimation of strength of associations [49]. Therefore it is possible that the associations reported here might also be underestimated. There is also the possibility that sedentary time was over estimated as actigraph does not measure sitting posture [33] but uses an activity count threshold that would have included some periods of quiet standing. In addition, this was a cross-sectional study and therefore some of the associations observed may be subject to cohort effects and other changes to lifestyle and health that may have impacted on accrual of bone mass other than the factors considered in this analysis. Finally, as almost 40% of cases had to be excluded due to accelerometry data not meeting the non-wear time criteria, there might be some differential bias. It is possible that non-wear time is associated with some direct or indirect risk factor for bone health and this could have influenced the results. Consequently, care must be taken in generalising the results.

## Conclusion

Screen-based SBs are negatively associated with femoral BMC in males and females and spinal BMC in females, independent of the time spent in moderate or vigorous activity. This deleterious effect is small and seems to be counteracted by engagement in strengthening activities and/or vigorous playtime in males and vigorous playtime in females. The pattern of intermittence between sedentary periods and activity appears to play a role in BMC with clustered short bouts of activity interspaced with long periods of sedentary behaviours appearing to be more beneficial than activities more evenly spread in time. The results suggest that only certain kinds of sedentary pursuits have a negative impact on bone health, which might be because they displace time that is otherwise spent in osteogenic activities. In addition, the results hint that it is the frequency of osteogenic activities such as vigorous playing and muscle strengthening activity, rather than volume of physical activity, that is important in limiting the deleterious effects of screen-based sedentary behaviours. Overall our results suggest that studying SB in young people is important, but it seems important to develop a more detailed classification of seated activity. In particular, studying the dynamics of sedentary behaviours might provide information about the most beneficial pattern of intermittence between osteogenic pursuits and period of rests for bone health. In turn, this could be used to guide activities in school.

## Competing interests

The authors declare that they have no competing interests.

## Authors’ contribution

All authors have substantially contributed to this work. SFMC and OM have performed the data processing and statistical analysis. SFMC and DAS have written the manuscript. All authors have edited, read and approved the final manuscript.

## Pre-publication history

The pre-publication history for this paper can be accessed here:

http://www.biomedcentral.com/1471-2458/14/4/prepub
